# Rosie en Español, a Chatbot-Delivered Intervention for Spanish-Speaking, US-Based Latina Mothers: Pilot Randomized Controlled Trial

**DOI:** 10.2196/101898

**Published:** 2026-10-22

**Authors:** Elizabeth M Norell, Francia Ximena Marin Gutierrez, Daniel Smolyak, Heran Mane, Xiaohe Yue, Neha Srikanth, Xin He, Thu T Nguyen, Jordan Boyd-Graber, Pankaj Dipankar, Quynh C Nguyen

**Affiliations:** 1 Department of Behavioral and Community Health, University of Maryland, 4200 Valley Drive, Suite 2242, College Park, MD, 20742, United States; 2 National Institute of Nursing Research, National Institutes of Health, 31 Center Drive, Room 5B03, Bethesda, MD, 20892, United States, 1 3014354322; 3 Department of Epidemiology and Biostatistics, University of Maryland, College Park, MD, United States; 4 Department of Computer Science, UMIACS, University of Maryland, College Park, MD, United States

**Keywords:** AI, chatbots, mental health, perinatal anxiety and stress, Latina mothers, randomized controlled trial

## Abstract

**Background:**

Latina pregnant women and mothers of infants in the United States experience elevated risk for perinatal anxiety, stress, and depression alongside persistent barriers to culturally and linguistically responsive care. AI-powered digital health tools offer a potentially scalable approach to address these gaps, yet evidence on their acceptability and effectiveness for this population remains limited.

**Objective:**

We aimed to evaluate the feasibility, acceptability, and preliminary outcome changes of Rosie, a Spanish-language AI-powered chatbot designed with and for Latina mothers, on maternal mental health and healthcareuse outcomes.

**Methods:**

We conducted a mixed methods pilot randomized controlled trial with 30 Spanish-speaking Latina participants who were pregnant or parenting an infant younger than 6 months. Participants were randomized 1:1 to the Rosie intervention or a comparison book club condition that provided monthly children’s board books. Surveys administered at baseline and 3-month follow-up assessed depression (Patient Health Questionnaire-9), anxiety (Generalized Anxiety Disorder-7), perceived stress (Perceived Stress Scale), and health care use. Quantitative analyses examined within- and between-group changes, and qualitative feedback assessed acceptability and user experience.

**Results:**

Baseline mental health scores were low in both groups; however, several baseline demographic differences were observed between study arms, including age, pregnancy vs parenting status, marital status, education, and country of origin. Over 3 months, participants in the Rosie group demonstrated statistically significant within-group reductions in anxiety (mean baseline 3.27, SD 2.89; mean change −2.00, SD 2.94; *P*=.02) and perceived stress (mean baseline 30.00, SD 5.53; mean change −12.20, SD 12.99; *P*=.003). Depression scores declined modestly in both groups, with no significant between-group differences. No significant differences were observed in maternal emergency department use or adherence to well-baby visits. Engagement with Rosie was high, and qualitative feedback indicated that participants found the chatbot useful and supportive.

**Conclusions:**

This pilot study suggests that a culturally and linguistically tailored AI-powered chatbot is feasible and acceptable among Latina mothers during the perinatal period. Although anxiety and perceived stress decreased within the Rosie group, no significant between-group differences were observed. Within-group reductions in anxiety and perceived stress among Rosie participants are preliminary and should be interpreted cautiously because the trial was not powered to detect between-group effects and no statistically significant between-group differences were observed. Larger, adequately powered trials are needed to evaluate effectiveness.

## Introduction

### Background

As digital health interventions powered by AI continue to proliferate, it is critical to evaluate their effectiveness in meeting stated clinical and public health goals, including careful examination of how well these tools work and for whom they work. Such evaluation is particularly important for populations that experience persistent health disparities and structural barriers to care. Latina pregnant women and mothers of infants in the United States experience elevated risk for perinatal mental health conditions alongside persistent barriers to culturally and linguistically responsive care. Digital health interventions for this population must therefore be intentionally designed to reflect community needs and lived experiences. In this study, we describe mixed methods pilot testing of Rosie, a newly developed AI-powered chatbot mobile app, designed with and for Latina mothers.

### Maternal and Child Health Disparities for Latina Women and Their Infants

Approximately 8.5% to 11% of women in the United States experience mental health disorders such as depression during pregnancy, and evidence indicates that Latina women face a disproportionate burden of perinatal mental health risk. A study of 3317 mothers residing in small cities and rural areas in Pennsylvania found that Latina mothers had 40% higher odds of postpartum depression compared to their White counterparts [1]. While prevalence estimates of perinatal depression among Latina women vary across studies, converging evidence suggests a marked increase over the past decade. Notably, a retrospective analysis of 442,308 births using electronic health records from Kaiser Permanente Southern California documented an increase in postpartum depression among Latina mothers from 8.9% in 2010 to 18.8% in 2021 [1. This 111.2% relative increase was nearly double the 61.5% increase observed among White mothers during the same period [1, highlighting widening racial and ethnic disparities in maternal mental health[2].

The elevated prevalence of mental health conditions during pregnancy among Latina women reflects the interaction of multiple structural, social, and cultural determinants of health. Socioeconomic disadvantages, limited access to health insurance, linguistic barriers, caregiving burden, and exposure to chronic stressors contribute to increased vulnerability during the perinatal period. Perinatal mental health conditions have consequences that extend beyond pregnancy, as maternal depression and anxiety have been associated with adverse infant outcomes, including preterm birth, low birth weight, and later cognitive and socioemotional developmental challenges [3,4]. Despite these risks, Latina women remain substantially less likely to seek or receive mental health care. A retrospective cohort study of 29,601 mothers enrolled in New Jersey’s Medicaid program found that Latina women were less likely than White women to access care for perinatal mental health symptoms [5], underscoring persistent gaps in diagnosis, referral, and treatment.

Psychosocial stressors related to immigration and discrimination further exacerbate maternal and child health disparities among Latina women. In studies of pregnant and postpartum Latina mothers, higher levels of acculturative stress and experiences of everyday discrimination, measured using validated self-report scales, have been associated with elevated symptoms of prenatal and postpartum depressive symptoms [6-8]. Beyond interpersonal stressors, restrictive immigration policies and heightened enforcement environments may intensify stress during a critical developmental period. For example, a study examining the 2008 Iowa immigration raid reported a 24% increase in the likelihood of preterm birth and low birth weight among infants born to Latina mothers following the raid. Similar adverse birth outcomes were documented following restrictive immigration policies enacted after the 2016 US presidential election [9,10]. Beyond birth outcomes, maternal experiences of discrimination may have longer-term biological implications for children. An observational study of 70 Latina mother-child dyads found that recent maternal exposure to racial discrimination was associated with proinflammatory gene expression profiles in children, suggesting intergenerational transmission of stress-related health risk [11].

In addition to structural stressors, cultural and linguistic barriers limit access to high-quality perinatal mental health care for Latina women. A persistent shortage of Spanish-speaking health care providers poses a significant challenge for monolingual Spanish-speaking mothers seeking timely and comprehensible health information [12]. Even when interpreter services are available, linguistic nuances and cultural framing of psychological distress may contribute to miscommunication and reduced quality of care. Furthermore, culturally rooted values such as *familismo* (prioritizing family) and *marianismo* (traditional female sex stereotypes), and stigma surrounding mental health treatment, framing psychological symptoms as personal weakness rather than medical conditions, may discourage disclosure of emotional distress [13]. As a result, symptoms of anxiety and depression may remain underrecognized and undertreated in clinical settings, particularly when compared to more visible physical health concerns [14,15].

Improving access to culturally and linguistically appropriate mental health education, screening, and support during pregnancy and the postpartum period has the potential to improve outcomes for both mothers and infants [16]. Rosie, the Chatbot, represents a novel technological innovation that is not only linguistically congruent and culturally responsive but also timely and accessible, with the potential to reduce informational barriers and support Latina mothers during the perinatal period. Rosie was designed primarily as an informational and educational support tool rather than a psychotherapeutic intervention. We hypothesized that providing timely, culturally and linguistically accessible, evidence-based information could reduce informational barriers and uncertainty during pregnancy and early parenting and, therefore, potentially influence anxiety and perceived stress.

### Maternal and Child Health Digital Health Interventions for Latina Women

While digital interventions are well-studied in the maternal and child health context, relatively few are tailored to and explicitly tested with Latina women [17]. These interventions span a range of technologies, from telephone [18] and internet-based [19] to virtual reality [20]. The aims of these interventions include assessing and treating postpartum mental health [17], promoting postpartum weight loss [21], and targeting the social determinants of health [22].

Approaches to reducing cultural and linguistic barriers also varied by intervention. Some interventions had a broad focus on minority racial/ethnic communities. For example, Herring et al [21] deployed the Healthy4Baby intervention, a program comprising interactive text messages and Facebook messages to promote weight-loss strategies, to Black and Latina mothers enrolled in Philadelphia’s Special Supplemental Nutrition Education Program for Women, Infants, and Children (WIC). Their approach to cultural tailoring included hiring a peer mentor coach who had previously completed the Healthy4Baby program and conducting regular check-ins, in addition to the interactive text messages. They found that the combination of digital and peer mentoring interventions prevented more than 3 kg of weight gain compared to a usual care group [21]. However, this intervention was conducted in English; thus, only participants with English fluency were included in the present study. In contrast, Platt et al [22] focused on Spanish-speaking mothers who were majority immigrants (to the United States), deploying a social determinant intervention comprising multiple virtual discussion sessions, surveys, and assistance with accessing food and nutrition resources (through both text messages and student volunteers). All sessions, surveys, and resources were provided in Spanish and administered by bilingual staff [22]. The intervention led to significant reductions in symptoms of depression and parental stress. Barrera et al [19] adapted their intervention to both English- and Spanish-speaking mothers, recruited from 23 different countries. Their intervention consisted of a fully automated internet-based course designed to encourage participants to adopt a healthy lifestyle for themselves and their infants, featuring text-based information, images, video or audio clips, and postlesson worksheets. They found that their intervention was not overall significantly effective in reducing postpartum depression compared to a brochure-only control but was significantly effective for those with the highest prenatal depression vulnerability [19].

We conducted a mixed methods pilot randomized controlled trial evaluating Rosie, a Spanish-language, AI-powered chatbot designed with and for Latina pregnant women and mothers of infants in the United States. The primary aims were to assess the feasibility and acceptability of the Rosie intervention, including participant engagement and user experience. Secondary aims were to descriptively evaluate the preliminary outcome changes of Rosie on maternal mental health outcomes, specifically depressive symptoms, anxiety, and perceived stress, as well as maternal and infant health care use, including emergency department (ED) use and well-baby visit adherence. Consistent with the exploratory nature of a pilot study, we hypothesized that participants randomized to the Rosie intervention, compared with those assigned to a book club comparison condition, would exhibit larger reductions in stress, anxiety, depressive symptoms, and ED use for their infants over the 3-month study period.

## Methods

### Setting

We conducted an experimental pilot study to assess the feasibility, acceptability, and appropriateness of Rosie for the target population. The pilot also served to test study protocols, software, and hardware functionality, and staff coordination, allowing for the identification and remediation of issues before scaling to a full randomized controlled trial. Pilot data were further used to improve the accuracy of Rosie’s responses by refining existing models, implementing fine-tuning mechanisms, and applying heuristic adjustments. The primary focus of the pilot was to evaluate access to required resources and the feasibility of implementing the intervention components and activities as planned. Implementation challenges and potential solutions related to intervention delivery and performance were systematically identified. A sample size of 30 participants was selected in accordance with National Institutes of Health definitions for phase 1 clinical trials, providing sufficient representation of the target population to test study procedures and elicit participant feedback on the Rosie application [23]. Pre- and postintervention assessments were conducted to qualitatively and quantitatively evaluate Rosie and to describe changes in key health outcomes, including postpartum depression and frequency of ED visits. Analyses are presented as preliminary findings and should be interpreted accordingly, as this pilot study was not powered to detect treatment-control differences and hypothesis testing was not the primary objective.

### Participant Recruitment and Study Design

#### Overview

Recruitment occurred through multiple channels, including social media platforms (Facebook [Meta], Instagram [Meta], and X [X Corp]), partnerships with community-based organizations, and in-person outreach at community events such as health fairs and family resource drives. Eligible participants included individuals aged 14 years or older who were either pregnant or parenting an infant younger than 6 months, fluent in Spanish, and self-identified as members of a racial or ethnic minority group. However, no participants younger than 18 years were enrolled in this study; the youngest enrolled participant was 20 years of age. Participants were randomly assigned to the intervention (Rosie) or comparison (book club) condition using a 1:1 allocation ratio, implemented by a study team member using a randomization table. Blinding was not feasible, and thus not used by participants or research staff.

#### Ethical Considerations

This study protocol was reviewed and approved by the Institutional Review Board at the University of Maryland (1556200) and was conducted in full compliance with institutional and federal standards for research involving human participants. All participants provided written (electronic) informed consent before completing baseline study procedures and engaging with study interventions.

Regarding privacy and confidentiality, study data were stored on secure, password-protected, and access-restricted servers. Analytic datasets were deidentified and stripped of direct personal identifiers before data processing and statistical analysis.

Regarding participant compensation, participants received a US $15 electronic gift card incentive for completing each of the 2 study surveys (baseline and 3-month follow-up; up to US $30 total). To incentivize engagement, participants randomized to the Rosie intervention arm also received US $0.50 per health-related question submitted to the chatbot, capped at a maximum of US $50. In addition, the top 20% most active Rosie users at the conclusion of the 3-month intervention received a curated set of children’s board books as an engagement prize

### Book Club Comparison Condition

Participants randomized to the book club comparison condition received 1 age-appropriate children’s board book per month for 3 months, for a total of 3 books. The book club was selected as a low-intensity active comparison condition intended to support participant retention and provide families with a meaningful parenting-related resource without exposing participants to the core components of the Rosie intervention. Specifically, book club participants did not receive access to AI-generated responses, personalized maternal or infant health information, source-linked health guidance, daily health tips, or interactive app features. This comparison condition was intended to account for some nonspecific aspects of study participation, including receiving materials from this study’s team, receiving a parenting-related resource, and experiencing a sense of support through participation in this study. However, the book club did not have access to several features unique to Rosie, including digital interaction, real-time question answering, personalized health guidance, or financial incentives tied to intervention use. As such, the book club served as a low-intensity active comparator rather than an attention-matched or fully equivalent intervention condition.

### Rosie Intervention Description

#### Overview

From a behavioral and clinical perspective, Rosie was designed to support maternal mental health by reducing uncertainty and informational barriers during pregnancy and early parenting. Participants could ask questions in Spanish at any time and receive conversational responses grounded in vetted maternal and infant health resources. The intervention was intended to provide maternal and child health information, anticipatory guidance, reassurance about common symptoms, and prompts to seek professional care when questions involved potentially urgent concerns. Unlike a static bilingual information repository, Rosie allowed participants to pose personally relevant questions in natural language, receive tailored responses with links to source materials, and access multiple app features including frequently asked questions (FAQs), videos, daily health tips, and evidence-grounded answer displays (Multimedia Appendix 1). Rosie was not designed to diagnose mental health conditions, provide psychotherapy, replace clinical care, or manage emergencies.

The system powering Rosie, the Chatbot, comprises 3 modules corresponding to 3 phases of answer construction: information retrieval, question-based information ranking, and answer generation. To ensure that our chatbot produces linguistically accurate, culturally attuned, and clinically reliable text, we constructed a repository from vetted health authorities (eg, CDC [Centers for Disease Control and Prevention], National Institutes of Health, FDA [Food and Drug Administration], AAP [American Academy of Pediatrics], ACOG [American College of Obstetricians and Gynecologists], and major academic medical centers). The core knowledge base comprises 122 institutional web sources, of which 49.2% (n=60) were natively authored Spanish-language resources (eg, CDC en Español, MedlinePlus en Español, and Salud Madre) and 50.8% (n=62) were curated English-language resources translated into Spanish. Translations were generated using Opus-MT (Helsinki-NLP/Marian NMT) [24,25] and validated by 3 native Spanish-speaking annotators who were public health professionals and researchers, with 97% of the 100 sampled documents rated as acceptable or strong. Annotations prioritized neutral Spanish (español neutro) to ensure intelligibility across participants from varied national backgrounds. In total, the corpus contains over four million indexed Spanish passages. Retrieval-augmented generation with sentence-level grounding scores was used to ensure clinical fidelity to source documents and communicate evidence support directly to users.

The retrieval and generation workflow operates through a multistage pipeline:

1. Dense retrieval: Each indexed passage is embedded into vector space using Contriever [26]. When a participant submits a query, the system encodes the question and retrieves the top 20 candidate passages based on cosine similarity.
2. Relevance reranking: A cross-encoder language model, multilingual-e5 [27], evaluates and reranks these candidates, selecting the top 10 most relevant passages.
3. Answer generation: A locally hosted large language model (Llama 3.1 8B [28] run on a university server rather than via third-party APIs) generates a conversational Spanish response conditioned strictly on the user query and the top 10 evidence passages.

#### Model Transparency and Interpretability

In a generative retrieval-augmented generation system such as Rosie, model transparency and explainability are established through explicit source provenance and sentence-level evidence verification rather than traditional feature attribution methods (eg, SHAP [Shapley additive explanations] or LIME [local interpretable model-agnostic explanations] applied to tabular risk scores). To communicate model faithfulness and mitigate hallucination risks [29,30], the system computes embedding similarity scores between each generated sentence and the underlying source evidence. High-similarity sentences are visually highlighted in the user interface, and direct URLs to authoritative source documents are displayed beneath the answer. This design aligns with emerging frameworks for retrieval-grounded clinical large language model evaluation and human-centered AI principles emphasizing transparency, trust, and auditability in health care [31]. More broadly, Rosie’s multimodule architecture and user-facing communication of response grounding are consistent with human-centered AI design principles emphasizing transparency, trust, usability, and safety in health care applications [32].

#### Participant Safety Procedures

Participant safety procedures were incorporated into onboarding, app messaging, crisis-response logic, and study oversight. Participants were informed that Rosie was an educational support tool and not a substitute for medical care, mental health treatment, diagnosis, emergency services, or clinical triage. The app instructed users to contact emergency services, their health care provider, a crisis hotline, or the nearest ED for suicidal ideation, thoughts of self-harm, severe psychological distress, obstetric warning signs, infant emergencies, or other urgent concerns.

Rosie included deterministic keyword-based detection for crisis and urgent clinical terms, including terms related to suicide, self-harm, abuse, severe depression, acute anxiety, bleeding, preeclampsia symptoms, fever, decreased fetal movement, and infant emergency symptoms. When such terms were detected, the system bypassed generative response generation and displayed a safety message directing the participant to emergency or crisis resources. These events also generated an alert to designated study staff for review and participant follow-up according to this study’s safety protocol. No study-related serious adverse events were reported during this pilot.

We used a continuous feedback process during this trial to identify any problems—such as gaps in the knowledge base for the chatbot, translation challenges, and broken links—fixing any problems as they were identified by participants’ quality ratings and any messages received from participants. As feedback was overwhelmingly positive, we maintained the key components of the app at the conclusion of this trial.

### Data Collection

Study data were collected using 2 self-administered online questionnaires, administered via Qualtrics, at baseline (preintervention) and at the 3-month follow-up (postintervention). The questionnaires assessed sociodemographic characteristics, maternal and infant health indicators, health care use, health-related behaviors, and participant feedback regarding the Rosie application. Survey completion was monitored throughout the data collection period to ensure completeness and data quality. Standard data management procedures were applied to identify missing responses, assess internal consistency, and resolve data inconsistencies before analysis. Survey data were subsequently integrated with intervention engagement metrics captured directly through the Rosie chatbot platform.

Validated self-report instruments were used to assess primary mental health outcomes. Depressive symptoms were measured using the Patient Health Questionnaire-9 (PHQ-9), a widely used 9-item scale assessing the frequency of depressive symptoms over the past 2 weeks. Items are rated on a 4-point Likert scale ranging from 0 (“not at all”) to 3 (“nearly every day”), yielding total scores from 0 to 27, with higher scores indicating greater symptom severity [33]. Anxiety symptoms were assessed using the Generalized Anxiety Disorder-7 (GAD-7), a 7-item measure evaluating the frequency of anxiety symptoms over the past 2 weeks using the same 4-point response scale, with total scores ranging from 0 to 21 [34]. Perceived stress was measured using the Perceived Stress Scale (PSS), which assesses the extent to which respondents perceive their lives as unpredictable, uncontrollable, and overloaded during the past month; items are summed to generate a total perceived stress score, with higher scores reflecting greater perceived stress [35]. All instruments have demonstrated strong reliability and validity across diverse populations, including Spanish-speaking samples. Health care use outcomes included self-reported maternal ED visits and adherence to recommended well-baby visits in accordance with American Academy of Pediatrics guidelines during the study period [36]. Participant feedback on the usability of the intervention, perceived helpfulness, and overall experience with Rosie was collected via postintervention survey items. Together, these data sources allowed for a comprehensive assessment of changes in mental health outcomes, health care use, and user engagement during this pilot trial.

### Statistical Analysis

Descriptive statistics were used to summarize participant characteristics and baseline measures by study group. Categorical variables were compared between groups using Fisher exact tests, and continuous variables were compared using Wilcoxon rank-sum tests to account for small sample size and nonnormal distributions. For primary mental health outcomes (depression [PHQ-9], anxiety [GAD-7], and perceived stress [PSS]), within-group pretest-posttest changes were assessed using paired *t* tests, and between-group differences in change scores were evaluated using independent sample *t* tests. For health care use outcomes, including maternal ED visits and well-baby visit adherence rate, between-group comparisons at posttest were conducted using Wilcoxon rank-sum tests. A sensitivity analysis was performed for depression outcomes to assess the influence of an outlier with a marked increase in PHQ-9 score from baseline to follow-up. Further sensitivity analyses were conducted to examine the role of pregnancy (vs parenting) status and marriage status in determining mental health outcomes (depression, anxiety, and perceived stress). Adjusted linear regression was used, with posttest mental health score as the outcome, Rosie vs book club group as a binary independent variable, and 3 covariates: pregnant (vs parenting), married (vs not married), and pretest mental health score. Heteroskedasticity-consistent SEs were used given the small sample size and nonnormality of model residuals. Due to sparse cell distributions across other demographic categories (eg, country of origin and educational strata), full multivariable adjustment across all baseline covariates was not performed to prevent model overfitting. Primary analyses remain unadjusted and should be interpreted as exploratory and hypothesis-generating.

All analyses were conducted with full awareness of group assignment, consistent with the unblinded pilot design. Statistical significance was assessed at a 2-sided α level of 0.05. Analytic sample sizes varied by outcome because of item-level missingness at follow-up. Denominators are therefore reported separately for each table and outcome. Given the pilot nature of this study and the limited sample size, analyses were exploratory and intended to assess preliminary outcome changes descriptively rather than to formally test hypotheses.

## Results

### Overview

The CONSORT (Consolidated Standards of Reporting Trials) diagram below summarizes our screening, randomization, baseline data collection, implementation of the intervention and control conditions, and 3-month follow-up data collection activities (Figure 1).

Table 1 summarizes baseline characteristics of the 30 participants randomized to the Rosie intervention group (n=15) or book club comparison group (n=15). Participants in both groups were predominantly Latina/Hispana, all identified as women, and most reported straight or heterosexual sexual orientation. Specifically, 93.3% (14/15) of participants in the Rosie group and 100% (15/15) in the book club group identified as Latina/Hispana.

Several descriptive differences between groups were observed, as expected in a small pilot randomized trial. A higher proportion of Rosie participants were pregnant at baseline compared with book club participants, whereas a higher proportion of book club participants were parenting an infant: 53.3% (8/15) of Rosie participants were pregnant, and 46.7% (7/15) were parenting, compared with 20% (3/15) pregnant and 80% (12/15) parenting in the book club group (*P*=.14). Educational attainment also varied descriptively between groups. In the Rosie group, the most common education level was high school graduate or General Educational Development (8/15, 53.3%), whereas in the book club group the most common education level was bachelor’s degree (6/15, 40%); this difference was not statistically significant (*P*=.23). Household income did not differ significantly between groups (*P*=.17), although income distributions varied across categories. Participants reported diverse countries of origin, most commonly El Salvador, Guatemala, Colombia, Honduras, and Mexico, with no statistically significant between-group difference in country of origin (*P*=.37).

Marital status differed descriptively between groups and approached conventional statistical significance (*P*=.05). Participants in the book club group were more often married than those in the Rosie group, whereas Rosie participants were more often unmarried but living with a partner. Age was the only baseline characteristic that differed significantly between groups (*P*=.02), with book club participants being older than Rosie participants; median age was 29.0 (IQR 25.5‐32.5) years in the Rosie group and 34.7 (IQR 30.0‐39.0) years in the book club group. Given the small pilot sample size, these baseline differences were interpreted descriptively and considered in sensitivity analyses and in the interpretation of outcome findings.

**Figure 1.**
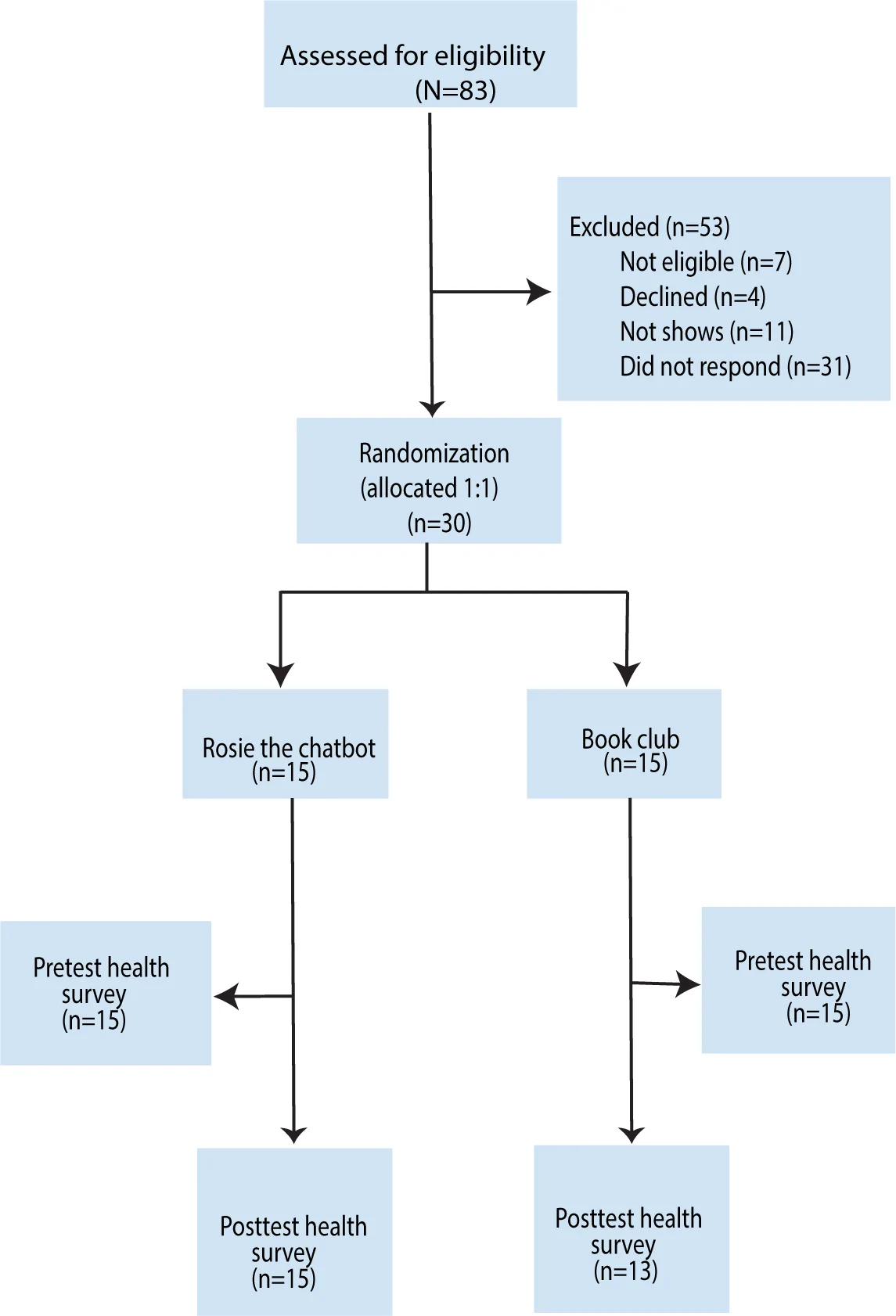
CONSORT (Consolidated Standards of Reporting Trials) diagram. Flow of participants through enrollment, allocation, follow-up, and analysis in the pilot randomized controlled trial. A total of 83 individuals were assessed for eligibility, of whom 30 were randomized 1:1 to either the Spanish Rosie chatbot intervention group (n=15) or the book club active comparison group (n=15). Follow-up rates reflect completion of baseline (pretest) and postintervention assessments, detailing exclusions and loss to follow-up across both study arms.

**Table 1.** Baseline demographic and clinical characteristics of Spanish-speaking Latina mothers in a pilot randomized controlled trial of the Rosie chatbot vs book club control (United States, May-October 2025).^a^

	Rosie (n=15)	Book club (n=15)	*P* value
Parenting status, n (%)			
Pregnant			.14
No	7 (46.7)	12 (80.0)	
Yes	8 (53.3)	3 (20.0)	
Parenting	12		
No	8 (53.3)	3 (20.0)	.14
Yes	7 (46.7)	12 (80.0)	
Race/ethnicity, n (%)			>.99
Latina/Hispana	14 (93.3)	15 (100.0)	
India Americana/Nativa de Alaska	1 (6.7)	0 (0.0)	
Gender identity, n (%)			>.99
Woman	15 (100.0)	15 (100.0)	
Sexual orientation, n (%)			
Straight or heterosexual	14 (93.3)	15 (100.0)	>.99
Prefer not to say	1 (6.7)	0 (0.0)	
Education, n (%)			.23
Less than high school	3 (20.0)	2 (13.3)	
High school graduate or GED^b^	8 (53.3)	3 (20.0)	
Associate’s degree	1 (6.7)	3 (20.0)	
Bachelor’s degree	2 (13.3)	6 (40.0)	
Master’s degree	1 (6.7)	1 (6.7)	
Household income (US $), n (%)			.17
0-30K	7 (46.7)	3 (20.0)	
30K-50K	5 (33.3)	8 (53.3)	
50K-75K	1 (6.7)	2 (13.3)	
75K-100K	0 (0.0)	2 (13.3)	
Other	2 (13.3)	0 (0.0)	
Country of origin, n (%)			.37
El Salvador	3 (20.0)	7 (46.7)	
Colombia	2 (13.3)	2 (13.3)	
Guatemala	3 (20.0)	1 (6.7)	
Honduras	2 (13.3)	1 (6.7)	
Mexico	2 (13.3)	1 (6.7)	
Ecuador	2 (13.3)	0 (0	
Nicaragua	0 (0.0)	2 (13.3)	
Estados Unidos	1 (6.7)	0 (0	
Peru	0 (0.0)	1 (6.7)	
Marital status, n (%)			.05
Married	4 (26.7)	11 (73.3)	
Never married	2 (13.3)	1 (6.7)	
Separated	2 (13.3)	0 (0.0)	
Not married, but living with a partner	7 (46.7)	3 (20.0)	
Age, median (IQR)	29.0 (25.5-32.5)	34.7 (30.0-39.0)	

a Data are derived from a 3-month pilot randomized controlled trial (conducted from May to October 2025 in the United States) evaluating Rosie, a Spanish-language AI-enabled conversational agent for maternal mental health. The study sample (n=30) comprised Spanish-speaking Latina mothers who were pregnant or parenting an infant younger than 6 months, randomized to either the Rosie intervention (n=15) or a book club control condition (n=15). Categorical variables are reported as frequency counts and percentages, n (%); continuous variables are presented as median and IQR. Between-group differences at baseline were evaluated using Fisher exact test for categorical variables and the Wilcoxon rank-sum test for continuous variables. *P* values <.05 were considered statistically significant.

b GED: General Educational Development.

### Health Outcomes

Table 2 compares changes in mental health and health care use outcomes between participants in the Rosie intervention group and the book club control group. At baseline, mean depression (PHQ-9) and anxiety (GAD-7) scores were low and comparable across groups. From pretest to posttest, depression scores decreased modestly in both groups (Rosie mean −0.67, SD 3.22; control mean −0.15, SD 2.18), with no significant between-group difference in change (*P*=.47) and no significant within-group changes. A sensitivity analysis was conducted for depression outcomes to assess the influence of a single Rosie participant whose PHQ-9 score increased markedly from pretest to posttest (4 to 12).

**Table 2.** Between-group and within-group differences in maternal mental health and health care use outcomes across study arms at 3-month follow-up.^a^

	Rosie (treatment group, n=15)	Book club (control group, n=13)	Between-group *P* value
Maternal Depression Scale, mean (SD)			
Pretest period	3.93 (3.82)	2.85 (2.18)	
Posttest period	3.27 (3.38)	2.69 (2.05)	
Pre- to posttest change	−0.67 (3.22)	−0.15 (2.18)	.47^b^
Within-group *P* value	.45^c^	.81^c^	
Maternal Anxiety Scale, mean (SD)			
Pretest period	3.27 (2.89)	3.92 (3.69)	
Posttest period	1.27 (1.69)	2.46 (2.41)	
Pre- to posttest change	−2.00 (2.94)	−1.46 (3.00)	.65^b^
Within-group *P* value	.02^c,d^	.11^c^	
Perceived Stress Scale, mean (SD)			
Pretest period	30.00 (5.53)	26.60 (11.48)	
Posttest period	17.80 (13.20)	22.23 (9.43)	
Pre- to posttest change	−12.20 (12.99)	−4.62 (14.91)	.18^b^
Within-group *P* value	.003^c,d^	.30^c^	
Maternal ED^e^ visits, n (%)			
0 visits	11 (73.3)	8 (61.5)	.54^f^
1 visit	3 (20.0)	2 (15.4)	
2 visits	1 (6.7)	3 (23.1)	
Pre- to posttest change, mean (SD)	0.07 (1.06)	0.00 (0.96)	.68^g^
Well-baby visit adherence rate (%), median (IQR)			
Posttest period	52 (33 to 75)^h^	94 (46 to 100)^i^	.23^j^
Pre- to posttest change	−19 (−53 to −3)^h^	−4 (−41 to 0)^i^	.52^k^

a Statistical analysis for 3-month pilot study of Rosie the AI-enabled chatbot conducted from May to October 2025 in the United States for maternal health among Latina mothers in the United States. Analysis compares Rosie intervention group to book club control group across 5 outcomes: depression, anxiety, stress, ED visits, and well-baby visit adherence. Sample sizes vary across outcomes because of item-level missingness at follow-up. Mental health outcomes include participants with both baseline and follow-up measures. Maternal ED use includes participants who completed the relevant follow-up use item. Well-baby visit adherence includes participants with available infant preventive care data. Percentages are calculated using the number of participants with available data for each outcome.

b For between-group comparison of pre- to posttest changes in depression, anxiety, and perceived stress scores, an independent-sample *t* test was conducted.

c For within-group comparison of pretest vs posttest in depression, anxiety, and perceived stress scores, a paired *t* test was conducted.

d *P*<.05.

e ED: emergency department.

f For between-group comparison of posttest outcomes for maternal ED visits, a Fisher-Freeman-Halton test was conducted, as participants reported either 0, 1, or 2 visits.

g For between-group comparison of pre- to posttest changes in maternal ED visits, a Mann-Whitney *U* test was conducted.

h n=10.

i n=12.

j For between-group comparison of posttest outcomes for well-baby visit adherence rate, a Wilcoxon rank-sum test was conducted, as the rates are not normally distributed.

k For between-group comparison of pre- to posttest changes in well-baby visit adherence rate, a Wilcoxon rank-sum test was conducted.

After excluding this participant, mean depression scores in the Rosie group decreased from 3.93 (SD 3.95) at pretest to 2.64 (SD 2.52) at posttest, corresponding to a larger mean prepost reduction of −1.29. The within-group change approached statistical significance (*P*=.07). However, the between-group difference in pre-post change between Rosie and the control group remained nonsignificant (*P*=.22).

Anxiety scores decreased in both groups, with a larger reduction observed in the Rosie group (mean −2.00, SD 2.94) compared with the control group (mean −1.46, SD 3.00); however, the between-group difference in change was not statistically significant (*P*=.65). Within-group analyses showed a significant reduction in anxiety in the Rosie group (*P*=.02), but not in the control group (*P*=.11).

Perceived stress scores also decreased in both groups from pretest to posttest. Within the Rosie group, the stress scores decreased significantly (*P*=.003) from pretest to posttest, with an average reduction of 12.20 (SD 12.99). Within the control group, the average reduction in stress score of 4.62 (SD 14.91) was not significant (*P*=.30). Between-group differences in stress reductions were not significant (*P*=.18).

The linear regression sensitivity analysis accounting for group differences in pregnancy status, marriage status, and age on mental health reported similar results (Multimedia Appendix 2). The coefficient for the Rosie intervention was negative across the 3 outcomes (depression mean −1.10, SD 0.83; anxiety mean −0.28, SD 1.12; perceived stress mean −6.23, SD 6.92), although none of the coefficients were significant (*P*>.05). The magnitude of these adjusted estimates is similar to the unadjusted between-group difference in change scores, suggesting that adjustment for age, pregnancy status, marital status, and baseline outcome values did not materially alter the estimated between-group difference.

There were no significant between-group differences in posttest maternal ED visits (median 0.00 in both groups; *P*=.54). In a sensitivity analysis, dichotomizing maternal ED use (any ED visit vs none), 4 of 15 participants in the Rosie intervention group and 5 of 13 participants in the book club control group reported at least one ED visit during the posttest period. Fisher exact test showed no significant difference between groups (odds ratio=1.72, *P*=.69), indicating that the likelihood of having any ED visit did not differ between the Rosie and control groups. Between-group differences for well-baby visit adherence rate were not statistically significant (*P*=.23), although median visit adherence rate was higher in the control group (94%) compared to the Rosie group (52%). Overall, while participants in the Rosie group demonstrated a significant within-group improvement in anxiety, no outcomes differed significantly between groups over this study’s period. Although this study was not powered to detect these differences, reporting them may be valuable for contextualization and hypothesis generation.

### Acceptability Findings

Table 3 presents statistics on Rosie use frequency and acceptability. More than 67% (10/15) of Rosie treatment group members used the Rosie question-and-answer feature at least once per week, on average, with 33.3% (5/15) of members using the feature at least every two days or daily. Rosie treatment group members, on average, asked 40.93 (SD 40.57) questions throughout this study, with the median member asking 27 questions (IQR 11.25‐54.75). Participants asked more questions in the early weeks of this study than in the later weeks (Figure 2). Only 1 participant, out of the 14 members who completed this section of the survey, reported issues with Rosie taking a long time to provide an answer or difficulties with updating the app. No participants had issues with storage space constraints or unexpected logouts. Participants reported high satisfaction with Rosie’s answers, with an average agreement score above 4 (1=strongly disagree, 5=strongly agree) across all metrics, including “high quality” (4.08, SD 1.26), “easy to understand” (4.54, SD 0.66), and overall satisfaction (4.54, SD 0.88).

**Table 3.** Acceptability and usability ratings among Latina mothers in the Rosie conversational AI intervention arm at 3-month follow-up (n=15; United States, 2025).^a^

Questions and response options	Value
How often do you use Rosie? At least... (n=15), n (%)	
Once per day	2 (13.3)
Once every 2 days	3 (20.0)
Once per week	5 (33.3)
Once per month	4 (26.7)
Never	1 (6.7)
Did you experience any of the following issues while using the app? (n=14), n (%)	
Rosie took a long time to provide an answer	1 (7.1)
Difficulties updating the app	1 (7.1)
Rosie took up too much storage space on my phone	0 (0.0)
Unexpected logouts	0 (0.0)
Please rate how much you agree with the following statements based on your thoughts and experiences (1=strongly disagree; 5=strongly agree; n=13), mean (SD)	
The answers I got to my health questions in the Rosie app were high quality	4.08 (1.26)
The answers from Rosie were easy to understand	4.54 (0.66)
The answers from Rosie were useful to me as a parent	4.62 (0.65)
I am satisfied with Rosie’s answers to my questions	4.54 (0.88)

a Data were collected at the 3-month postintervention follow-up from a pilot randomized controlled trial evaluating Rosie, an AI-enabled Spanish-language chatbot on maternal health among Latina pregnant women and mothers of infants younger than 6 months in the United States (2025). Sample sizes vary across survey sections due to item-level completion: use frequency (n=15), technical or app issues (n=14), and satisfaction statement ratings (n=13). Satisfaction statements were rated on a 5-point Likert scale ranging from 1 ("strongly disagree") to 5 ("strongly agree") and are presented as mean (SD).

**Figure 2.**
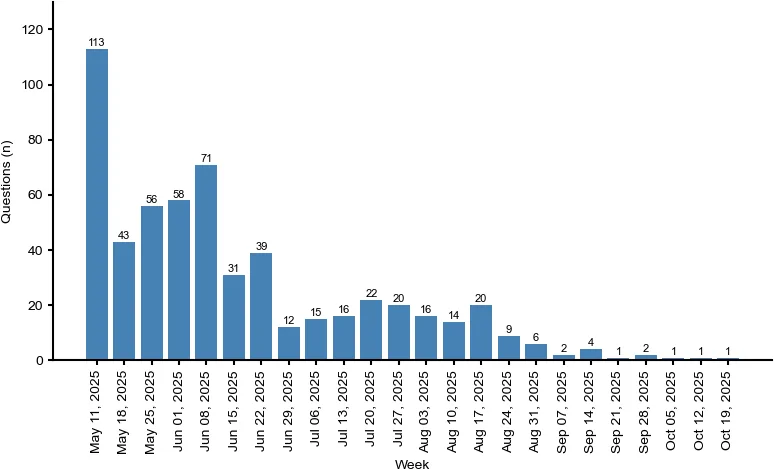
Weekly volume of health-related questions submitted to the Rosie conversational AI chatbot across the 3-month pilot randomized controlled trial (n=15; United States, May to October 2025). Aggregate weekly query submissions are displayed across the 12-week intervention period.

Participants also reported frequent use of specific features within the Rosie app and chatbot (Tables 4 and 5). At least half of the time, 28.5% (4/14) of participants reported clicking on Rosie’s answer to read more information at the source website, and half of participants (7/14) reported providing ratings for Rosie’s answers. At least 64.3% (10/14) of participants reported using each of Rosie’s nonchatbot features at least a few times, including Rosie’s video library, FAQ section, and daily health tips (which 4 participants used at least daily).

**Table 4.** Self-reported frequency of mobile app feature use among Rosie intervention participants at 3-month follow-up (n=14; United States, 2025).^a^

How often did you...	Most of the time, n (%)	Half of the time, n (%)	A few times, n (%)	Never, n (%)
Click on Rosie’s answer to read more information at the source website	3 (21.4)	1 (7.1)	9 (64.3)	1 (7.1)
Provide a rating for Rosie’s answers	6 (42.9)	1 (7.1)	5 (35.7)	2 (14.3)

a Data from the 3-month postintervention survey in a pilot randomized controlled trial evaluating Rosie among Spanish-speaking Latina mothers in the United States (2025). Denominators reflect the 14 intervention participants who completed the feature-use survey module. Values are reported as frequency counts and percentages. Evaluated features include external source citation verification links, answer star ratings, educational video repository, frequently asked questions, daily health tips, and the points or incentive reward tracker.

**Table 5.** Self-reported frequency of mobile app feature use among Rosie intervention participants at 3-month follow-up (n=14; United States, 2025).^a^

How often did you...	Multiple times a day, n (%)	Once daily, n (%)	Weekly, n (%)	A few times, n (%)	Never, n (%)
Use Rosie’s video library	1 (7.1)	1 (7.1)	3 (21.4)	4 (28.6)	5 (35.7)
Use the FAQ^b^ section	1 (7.1)	0 (0.0)	2 (14.3)	8 (57.1)	3 (21.4)
Read the Daily Syllabus or review daily health tips?	2 (14.3)	2 (14.3)	3 (21.4)	4 (28.6)	3 (21.4)
Check your points earned by asking questions in Rosie?	1 (7.1)	0 (0.0)	3 (21.4)	7 (50.0)	3 (21.4)

a Data from the 3-month postintervention survey in a pilot randomized controlled trial evaluating Rosie among Spanish-speaking Latina mothers in the United States (2025). Denominators reflect the 14 intervention participants who completed the feature-use survey module. Values are reported as frequency counts and percentages. Evaluated features include external source citation verification links, answer star ratings, educational video repository, frequently asked questions, daily health tips, and the points or incentive reward tracker.

b FAQ: frequently asked questions.

Ratings for individual Rosie answers were mostly positive, as seen in Figure 2. The rating system ranged from 1 star (bad) to 5 stars (good). Of 305 ratings provided, 92.3% (n=281) were positive ratings (4 or 5 stars), with an average rating of 4.73 (SD 0.81). Of the 14 participants who provided at least one rating, only 3 participants gave at least one negative rating (3 or fewer stars). Participants provided more ratings in the early weeks of this study than in the later weeks (Figure 3).

**Figure 3.**
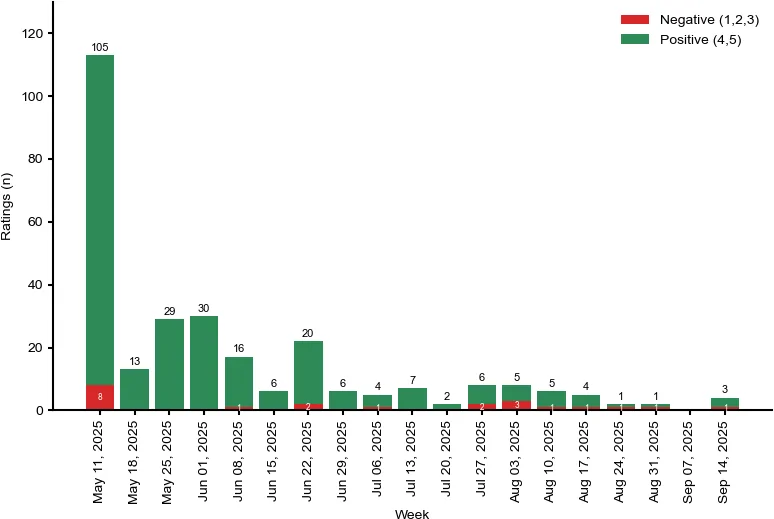
Weekly distribution of positive and negative participant ratings for Rosie chatbot responses across the 3-month pilot randomized controlled trial (n=15; United States, May to October 2025). Stacked weekly counts of user quality ratings for individual chatbot answers. Ratings were submitted on a 1- to 5-star scale and categorized as positive (green bars: 4 or 5 stars; 92.3%, n=281) or negative (red bars: 1, 2, or 3 stars; 7.7%, n=24) across a total of 305 submitted ratings (mean score: 4.73, SD 0.81). Rating activity was highest during initial onboarding weeks and mirrored the overall weekly question submission trends.

Participants in the control group also indicated general satisfaction with the book club (Table 6). With an average agreement score above 4.5 (1=strongly disagree, 5=strongly agree) across all metrics, participants agreed that the books were of good quality (4.46, SD 1.20), helpful during pregnancy or parenting (4.46, SD 0.66), and would recommend the book club to other parents (4.77, SD 0.44).

**Table 6.** Participant satisfaction and acceptability ratings for the book club comparison group at 3-month follow-up (n=13; United States, 2025).^a^

Please rate how much you agree with the following statements based on your thoughts and experiences (1=strongly disagree; 5=strongly agree)	Score, mean (SD)
The books I received were of good quality	4.46 (1.20)
The content of the books I received is a good match for my baby’s needs	4.46 (0.78)
The books were helpful to me during my pregnancy or parenting my infant	4.46 (0.66)
The books were enjoyable to me during my pregnancy or parenting my infant	4.54 (0.66)
Participating with the book club was easy for me	4.69 (0.48)
I would recommend the book club to other parents	4.77 (0.44)

a Data are reported from postintervention follow-up surveys administered to Spanish-speaking Latina pregnant women and mothers of infants randomized to the book club comparison condition during a 3-month pilot randomized controlled trial in the United States (2025). Participants in this condition received 1 age-appropriate children’s board book per month for 3 months (N=15 allocated; n=13 completed the posttest acceptability survey). Items were rated on a 5-point Likert scale: 1=strongly disagree, 2=disagree, 3=neutral or neither agree nor disagree, 4=agree, and 5=strongly agree. Higher scores indicate greater satisfaction, perceived usefulness, and acceptability.

### Qualitative Findings

Regarding the Rosie group, moms in the Rosie group actively engaged with the chatbot by asking questions (Table 7), using the FAQ, forum, and video pages. Their engagement was evidenced by the number of points they accumulated by asking Rosie the chatbot questions, which were then translated into compensation. When asked about feedback in the posttest survey, only 1 mom provided additional feedback about her experience in the Rosie group. She said, “very nice application, very useful, it helped me a lot.”

**Table 7.** Mixed methods joint display of quantitative mental health changes and illustrative user queries among Latina mothers using the Rosie chatbot.^a^

Health outcome^b^	Outcome change^c^ (pretest vs posttest)	Related questions asked by users
↓^d^ Maternal depression	−0.67 (95% CI −2.51 to 1.18); T-critical: 2.14; df: 14	“Hola rosi me está costando mucho producir leche materna” “Hi Rosi, I’m having a really hard time producing breast milk.” “Hola en qué momento del embarazo se quitan los dolores de cabeza” “Hi, at what stage of pregnancy do headaches go away?” “Síntomas de depresión post parto” “Symptoms of postpartum depression” “Que es depresión posparto?” “What is postpartum depression?” “Cómo quitar los cólicos del bebe” “How to relieve a baby’s colic”
↓^d^ Maternal anxiety	−2.00 (95% CI: −3.69 to −0.31); T-critical: 2.14; df: 14	“Que puedo tomar yo de mama para la ansiedad” “What can I take for anxiety while breastfeeding?” “Me da mucha ansiedad de comer después del parto” “I feel a lot of anxiety about eating after giving birth.” “Rosi mi bebé de 3 meses ya quiere empezar a gatear es normal?” “Rosi, my 3-month-old baby already wants to start crawling—is that normal?” “Es normal que mi bebé de 3 meses no vaya defecar en al noche” “Is it normal for my 3-month-old baby not to have a bowel movement at night?” “Porque siento como animalitos en la garganta” “Why does it feel like there are little critters in my throat?” “Cuántas veces se tiene que mover el bebé a las 34 semanas” “How many times should the baby move at 34 weeks?” “Es normal tener inflamada la cara en los últimos días de embarazo” “Is it normal to have a swollen face during the final days of pregnancy?”
↓^d^ Perceived stress	−12.20 (95% CI: −19.65 to −4.75); T-critical: 2.14; df: 14	“Descalcificación durante el embarazo” “Calcium depletion during pregnancy” “Cómo se puede evitar la preclancia” “How to prevent preeclampsia” “Si me duele a un lado del estimator que tengo que hacer” “What should I do if I have pain on one side of my abdomen?” “Puede una infección urinaria afectar al bebé durante el embarazo?” “Can a urinary tract infection affect the baby during pregnancy?” “Puedo quedar embarazada después de dar a luz” “Can I get pregnant again shortly after giving birth?” “Cómo saber si algo que comí le cayó mal a mi bebé en la leche materna” “How to tell if something I ate upset my baby through my breast milk” “Porque mis senos me duelen cuando sale leche” “Why do my breasts hurt when milk comes out?” “Porque me pega mucha hambre” “Why do I feel so extremely hungry?” “Por qué se cae el cabello al dar pecho” “Why does hair loss occur while breastfeeding?” “Que puedo comer si mi estomago me duele y no soporta nada” “What can I eat if my stomach hurts and can’t keep anything down?”
Maternal ED^e^ visits	—^f^	“Como controlar diabetes gestacional” “How to manage gestational diabetes” “Hinchazón en los pies durante el embarazo” “Swollen feet during pregnancy” “Síntomas de preeclampsia sin presión que sobrepasa de 128/84” “Symptoms of preeclampsia without blood pressure exceeding 128/84” “Por qué me duele el cóccix cuando me siento en las 38 semanas” “Why does my tailbone hurt when I sit at 38 weeks?” “Accidentes de auto en el embarazo?” “Car accidents during pregnancy?” “Que pasa cuando baja sangre en la 37 semana de embarazo” “What does it mean when you have vaginal bleeding at 37 weeks of pregnancy?” “Fiebre durante la semana 36 de embarazo” “Fever during the 36th week of pregnancy”
Well-baby visits	—^f^	“Porque mi bebé tiene los ojos hinchados y alrededor rojo y seco” “Why are my baby’s eyes swollen, with the skin around them red and dry?” “Hasta qué [nivel] de temperatura de la bebé tengo que llevarla a emergencies” “At what temperature should I take my baby to the emergency room?” “Reacciones de bebé al tener vacuna de dos meses” “Baby’s reactions to the two-month vaccinations” “Rosi, cuánto pesa un bebé de 4 meses?” “Rosi, how much does a 4-month-old baby weigh?”

a Data reflect the intervention arm (n=15) of a 3-month mixed methods pilot randomized controlled trial evaluating Rosie, a Spanish-language conversational AI chatbot, among Spanish-speaking Latina pregnant women and mothers of infants younger than 6 months in the United States (2025). Qualitative queries were submitted directly in Spanish via the chatbot interface and have been paired with verified English translations.

b Maternal depression was assessed using the Patient Health Questionnaire-9 (score range 0-27); maternal anxiety was assessed using the Generalized Anxiety Disorder-7 (score range 0-21); and perceived stress was assessed using the Perceived Stress Scale (score range 0-40).

c Pre- to postintervention mean change score calculated as posttest minus pretest score. Pretest-posttest within-group changes were assessed using a 2-tailed paired *t* test.

d ↓ (downward arrow) indicates a decrease (symptom reduction) in the measured score from baseline to 3-month follow-up.

e ED: emergency department.

f Not applicable.

Regarding the book club, mothers in the control group described the children’s books as meaningful and relevant to their identities and family contexts. Several participants emphasized the value of receiving inclusive books, particularly those available in their primary language. As 1 mother stated, “I thought it was wonderful to receive inclusive books made for children and in my language.”

Mothers also reported that the books were developmentally appropriate for their infants, noting that the colors and illustrations were engaging for their babies’ current developmental stage. One participant explained, “The colors and figures are eye-catching for the age my baby is now.” Additionally, participants described observing their infants actively engaging with the books during shared reading. Mothers reported behaviors such as visual tracking and vocalization, which they interpreted as indicators of early learning and interaction. One mother shared, “He follows the images with his eyes, and while we say the names of each country and interact with him while reading the book, he babbles along with us.” Overall, mothers reported positive experiences with the book club, describing it as an enjoyable, interactive activity that engaged the entire family.

## Discussion

### Principal Findings

This mixed methods pilot study examined the acceptability and preliminary outcome changes of Rosie, an AI-powered, Spanish-language chatbot designed with and for Latina pregnant women and mothers of infants in the United States. The pilot supports feasibility and acceptability and initial evidence of improvement in anxiety and perceived stress for Rosie users. It was not powered to detect and did not establish comparative efficacy in anxiety and perceived stress to an active control, but rather to inform power calculations for the larger trial. Baseline mental health scores were low in both groups. Over 3 months, participants in the Rosie group demonstrated statistically significant within-group reductions in anxiety and perceived stress; however, between-group differences in changes in depression, anxiety, and perceived stress were not statistically significant. No statistically significant between-group differences were observed in maternal ED use or well-baby visit adherence. The book club comparison condition may also have conferred nonspecific benefits, including perceived support from study participation, enjoyment, parent-child interaction, and access to developmentally appropriate materials in Spanish. These benefits may have contributed to improvements observed in the comparison group and may have reduced group differences. Therefore, the absence of statistically significant between-group differences should be interpreted in light of both the small sample size and the active nature of the comparison condition.

The observed reduction in anxiety and stress among Rosie users aligns with prior research indicating that digital mental health interventions may be especially effective in addressing anxiety-related symptoms, which are often responsive to psychoeducation, reassurance, and timely access to information [37-39]. Rosie’s conversational format and on-demand availability may have helped alleviate uncertainty and stress associated with pregnancy and early parenting, particularly in contexts where access to culturally and linguistically appropriate care is limited. While depression scores decreased modestly in both groups, these changes were not statistically significant. Sensitivity analyses suggested that depression outcomes in the Rosie group may be influenced by individual variability, highlighting the importance of larger samples and longer follow-up periods in future trials.

No significant differences were observed between groups in maternal ED use or well-baby visit adherence. These findings may reflect the short duration of follow-up, low overall rates of ED use, and the fact that health care use behaviors are influenced by structural factors such as insurance coverage, transportation, and clinic availability [40,41] that may not be readily modified by an informational digital intervention alone. Nevertheless, the lack of adverse effects and comparable use across groups supports the safety and appropriateness of the Rosie intervention in this pilot phase.

Quantitative and qualitative feedback indicated high acceptability of Rosie among participating mothers. Participants actively engaged with the chatbot by asking questions and using multiple app features, including FAQs, videos, and forums. Although open-ended qualitative feedback was limited, the available responses were uniformly positive, with participants describing the app as useful and helpful. The observed engagement is particularly encouraging given documented challenges in sustaining participation in digital health interventions [42,43].

Rosie’s acceptability likely reflects its intentional design features, including Spanish-language delivery, culturally responsive content, and grounding in trusted health information sources. Transparency about evidence sources and visual indicators of response grounding may have further contributed to user trust, an essential component for AI-enabled health tools, especially in communities that have historically experienced medical mistrust [44,45]. The continuous feedback process used during the pilot also allowed the research team to quickly identify and resolve technical and content-related issues, which may have further enhanced user experience and engagement.

Participant interaction patterns provide additional insight into the informational and emotional support needs of Latina pregnant women and mothers of infants. Mothers frequently asked about pregnancy symptoms and safety, infant feeding and digestion, maternal exposures during pregnancy or breastfeeding, infant illness and treatment, and developmental milestones. These topics reflect common areas of uncertainty during pregnancy and early parenting, particularly when timely access to linguistically concordant care is limited. Many prompts were phrased as short topics, symptom descriptions, or situation statements rather than complete questions, suggesting that users may engage with Rosie conversationally and informally similar to how they might ask a trusted family member, peer, or clinician for guidance. Reassurance-seeking formulations, such as whether a symptom was “normal” or whether a situation required emergency care, were also common. This pattern suggests that Rosie may have functioned not only as an information source but also as a tool for reassurance, normalization, and decision support.

These interaction patterns informed priorities for refinement of the platform. High-frequency topics highlighted the need to expand and further tailor content related to infant feeding, breastfeeding and milk supply, spit-up and vomiting, constipation, pregnancy warning signs, postpartum recovery, vaccines, and developmental milestones. The phrasing of queries also underscored the importance of improving Rosie’s ability to interpret short, misspelled, colloquial, or mixed-language prompts. In addition, questions about urgent symptoms, including bleeding, preeclampsia symptoms, decreased fetal movement, infant fever, vomiting, diarrhea, and car accidents during pregnancy, reinforced the need for clear safety guardrails, source-linked guidance, and escalation language directing users to clinical or emergency care when appropriate. Future development will focus on tailoring responses more closely to pregnancy stage, infant age, feeding method, and symptom severity while preserving Rosie’s role as an educational support tool rather than a replacement for clinical care.

Overall, this study has several strengths, including its community-informed design, focus on an understudied and underserved population, and use of a mixed methods approach to assess both outcomes and user experience. The pilot randomized design enabled a preliminary comparison between intervention and control conditions while also testing study procedures, technology functionality, and recruitment strategies in preparation for a larger trial. Additionally, Rosie’s architecture, grounded in vetted Spanish-language health sources and designed to mitigate hallucinations, represents a responsible application of generative AI in health care.

### Comparison With Prior Work

The results of this pilot study should be interpreted within the broader context of persistent maternal mental health disparities affecting Latina women in the United States. Structural stressors including immigration-related fears, discrimination, language barriers, and limited access to culturally concordant care contribute to elevated risk for perinatal anxiety, stress, and depression, while simultaneously limiting engagement with traditional mental health services [46-49]. Rosie represents an innovative attempt to address some of these barriers by offering linguistically accessible, on-demand educational support during a critical developmental period. However, whether Rosie can be implemented cost-effectively or at scale requires further evaluation in larger effectiveness and implementation studies.

Compared with prior digital health interventions for Latina mothers, which often rely on static content or clinician-mediated delivery [17,39,50], Rosie leverages generative AI to provide personalized, interactive responses in real time. The observed within-group improvements in anxiety and stress suggest that such an approach may be particularly well suited to addressing the informational and emotional needs that arise unpredictably during pregnancy and early parenting.

### Limitations

Limitations should also be noted. The small sample size limited statistical power to detect between-group differences and precluded subgroup analyses. Baseline levels of depression and anxiety were relatively low, potentially resulting in floor effects that constrained observable change and limiting our ability to assess outcomes among participants with clinically meaningful symptoms. In addition, despite randomization, the small pilot sample yielded baseline imbalances between study arms, including maternal age, pregnancy vs parenting status, marital status, educational attainment, and country of origin. Although only maternal age reached conventional statistical significance, these characteristics may be associated with mental health trajectories, health care use, and engagement with digital tools. Adjusted sensitivity analyses were conducted for selected covariates, but the small sample size limited the number of variables that could be included without overfitting. Therefore, observed within-group reductions in anxiety and perceived stress should be interpreted cautiously and should not be attributed causally to the intervention. For the control group, the receipt of books may itself have provided benefits through parent-child interaction, education, enjoyment, or perceived support. These nonspecific benefits may have reduced between-group differences; however, this study was neither powered for nor intended to detect statistically significant differences, as it is a pilot study.

Outcomes were self-reported and assessed over a relatively short follow-up period, which may not capture longer-term effects on mental health or health care use. Qualitative feedback from Rosie users was limited in depth, restricting insight into specific features or mechanisms driving perceived usefulness. Finally, participants received financial incentives tied to chatbot use, which may have influenced engagement patterns and limited generalizability to real-world implementation settings. Engagement findings should be interpreted cautiously because Rosie participants received compensation directly linked to the number of chatbot questions submitted, with additional rewards for the most active users. This incentive structure may have increased question volume and frequency of use and limits conclusions about intrinsic engagement in routine deployment.

Finally, Rosie’s generalizability is bounded by its development context, which was specifically tailored to Spanish-speaking Latina mothers navigating the United States health care system. Implementing this conversational AI framework in different linguistic, cultural, or health care settings would require substantial adaptation across three core areas: (1) linguistic adaptation, including expert-validated translation pipelines and tuning for local dialects and health literacy; (2) cultural adaptation, involving community-engaged co-design to align with local maternal-infant care beliefs and psychosocial norms; and (3) health system adaptation, rebuilding the retrieval repository around local clinical practice guidelines and regional emergency and health care referral pathways rather than US-centric standards.

### Conclusions

Despite these limitations, findings from this pilot study provide important guidance for future research and intervention development. Larger, fully powered randomized controlled trials are needed to evaluate Rosie’s effectiveness on maternal mental health outcomes, health care use, and infant health indicators over longer follow-up periods. Future studies should also examine differential effects by baseline mental health risk, immigration-related stressors, language preference, and prior access to care. Incorporating richer qualitative methods, such as interviews or focus groups, could further elucidate user experiences and inform continued refinement of chatbot content and functionality. Future trials also should either balance engagement-related incentives across conditions or evaluate Rosie under implementation conditions in which chatbot use is not directly compensated.

From a practice perspective, Rosie highlights the potential role of AI-enabled, culturally responsive digital tools as a complement, not a replacement, for traditional perinatal care. Such tools may be particularly valuable in safety-net and community-based settings where demand for Spanish-speaking providers exceeds supply. Thoughtful integration with clinical workflows, community organizations, and public health programs may enhance reach and sustainability while ensuring ethical and equitable use of AI technologies.

This pilot study demonstrates that Rosie, an AI-powered Spanish-language chatbot designed for Latina pregnant women and mothers of infants, is feasible and acceptable in a small, randomized pilot trial. Significant within-group reductions in anxiety and perceived stress were observed among Rosie participants; however, not statistically significant between-group differences were observed, and findings should be interpreted as exploratory and hypothesis-generating. Larger, adequately powered randomized controlled trials are needed to determine whether Rosie improves maternal mental health, health care use, or infant health outcomes. As AI continues to be integrated into digital health, community-engaged and culturally grounded approaches such as Rosie will be essential to ensuring that technological innovation advances, rather than exacerbates, health disparities.

## Supplementary material

10.2196/101898 Multimedia Appendix 1 Spanish Rosie mobile app figure.

10.2196/101898 Multimedia Appendix 2 Rosie vs control group differences in mental health outcomes, adjusting for pregnancy and marital status.

10.2196/101898 Checklist 1 CONSORT checklist.

## Abbreviations

AAP: American Academy of Pediatrics
ACOG: American College of Obstetricians and Gynecologists
CDC: Centers for Disease Control and Prevention
CONSORT: Consolidated Standards of Reporting Trials
ED: emergency department
FAQ: frequently asked questions
FDA: Food and Drug Administration
GAD-7: Generalized Anxiety Disorder-7
LIME: local interpretable model-agnostic explanations
PHQ-9: Patient Health Questionnaire-9
PSS: Perceived Stress Scale
SHAP: Shapley additive explanations
WIC: Women, Infants, and Children
