# Supplementary material for: Rosie en Español, a Chatbot-Delivered Intervention for Spanish-Speaking, US-Based Latina Mothers: Pilot Randomized Controlled Trial
Source: JMIR Form Res. 2026 Oct 22;10:e101898. doi: 10.2196/101898 (PMC13737348; doi:10.2196/101898)
Supplement: Supplementary file 2 [file formative-v10-e101898-s001.docx]

**Figure S1.** Spanish Rosie mobile application.

Panel left to right: A. Login page where users can log in using email or Apple ID/Android ID. B. Rosie chat page, where users can ask Rosie a health question. C. An example of Rosie’s response to a health question with web sources displayed and options for users to rate the response. D. Users who choose to provide a rating of Rosie’s response have the additional option of providing reasons for the rating. E. Daily tips page, F. Frequently asked questions (FAQ), G. Video library, H. Discussion forum, I. Profile page.
