# Supplementary material for: Rosie en Español, a Chatbot-Delivered Intervention for Spanish-Speaking, US-Based Latina Mothers: Pilot Randomized Controlled Trial
Source: JMIR Form Res. 2026 Oct 22;10:e101898. doi: 10.2196/101898 (PMC13737348; doi:10.2196/101898)
Supplement: Supplementary file 3 [file formative-v10-e101898-s002.docx]

| **Table S1.** Rosie versus control group differences in mental health outcomes, adjusting for pregnancy and marital status. | | | |
| --- | --- | --- | --- |
| Covariate | **Maternal Depression**  β (SE) | **Maternal Anxiety**  β (SE) | **Perceived Stress**  β (SE) |
| Rosie (treatment group)  vs Book Club (control) | -1.10 (0.83) | -0.28 (1.12) | -6.23 (6.92) |
| Pregnancy status (reference=parenting) | 0.96 (1.30) | -1.30 (1.00) | -0.50 (6.38) |
| Marital status (reference=unmarried) | -0.21 (0.83) | 0.87 (1.15) | 2.40 (9.00) |
| Age | -0.16 (0.11) | -0.02 (0.06) | -0.54 (0.50) |
| Outcome baseline | 0.36* (0.15) | 0.33* (0.15) | 0.02 (0.38) |
| Results of regression-based sensitivity analysis of the effect of differences in pregnancy status and marital status between the Rosie (intervention) and book club (control) groups. Significance (*p* < 0.05) is indicated by *, and the β (regression coefficient) and SE (standard error) for each covariate is provided. | | | |
